# Pharmacodynamics of zoliflodacin plus doxycycline combination therapy against *Neisseria gonorrhoeae* in a gonococcal hollow-fiber infection model

**DOI:** 10.3389/fphar.2023.1291885

**Published:** 2023-12-07

**Authors:** Susanne Jacobsson, Daniel Golparian, Joakim Oxelbark, Fabian Y. S. Kong, Renata Maria Augusto Da Costa, Francois Franceschi, David Brown, Arnold Louie, George Drusano, Magnus Unemo

**Affiliations:** ^1^ WHO Collaborating Centre for Gonorrhoea and Other STIs, National Reference Laboratory for Sexually Transmitted Infections, Department of Laboratory Medicine, Faculty of Medicine and Health, Örebro University, Örebro, Sweden; ^2^ Division of Clinical Chemistry, Department of Laboratory Medicine, Faculty of Medicine and Health, Örebro University, Örebro, Sweden; ^3^ Centre for Epidemiology and Biostatistics, Melbourne School of Population and Global Health, University of Melbourne, Melbourne, VIC, Australia; ^4^ Global Antibiotic Research and Development Partnership (GARDP), Geneva, Switzerland; ^5^ College of Medicine, Institute for Therapeutic Innovation, University of Florida, Orlando, FL, United States; ^6^ Institute for Global Health, University College London (UCL), London, United Kingdom

**Keywords:** *Neisseria gonorrhoeae*, hollow-fiber infection model, zoliflodacin, doxycycline, pharmacodynamics, antimicrobial treatment, pharmacokinetics

## Abstract

Antimicrobial resistance in the sexually transmitted bacterium *Neisseria gonorrhoeae* is compromising the management and control of gonorrhea globally. Optimized use and enhanced stewardship of current antimicrobials and development of novel antimicrobials are imperative. The first in class zoliflodacin (spiropyrimidinetrione, DNA Gyrase B inhibitor) is a promising novel antimicrobial in late-stage clinical development for gonorrhea treatment, i.e., the phase III randomized controlled clinical trial (ClinicalTrials.gov Identifier: NCT03959527) was recently finalized, and zoliflodacin showed non-inferiority compared to the recommended ceftriaxone plus azithromycin dual therapy. Doxycycline, the first-line treatment for chlamydia and empiric treatment for non-gonococcal urethritis, will be frequently given together with zoliflodacin because gonorrhea and chlamydia coinfections are common. In a previous static *in vitro* study, it was indicated that doxycycline/tetracycline inhibited the gonococcal killing of zoliflodacin in 6-h time-kill curve analysis. In this study, our dynamic *in vitro* hollow-fiber infection model (HFIM) was used to investigate combination therapies with zoliflodacin and doxycycline. Dose–range experiments using the three gonococcal strains WHO F (susceptible to relevant therapeutic antimicrobials), WHO X (extensively drug-resistant, including ceftriaxone-resistant; zoliflodacin-susceptible), and SE600/18 (zoliflodacin-susceptible strain with GyrB S467N substitution) were conducted simulating combination therapy with a single oral dose of zoliflodacin 0.5–4 g combined with a doxycycline daily oral dose of 200 mg administered as 100 mg twice a day, for 7 days (standard dose for chlamydia treatment). Comparing combination therapy of zoliflodacin (0.5–4 g single dose) plus doxycycline (200 mg divided into 100 mg twice a day orally, for 7 days) to zoliflodacin monotherapy (0.5–4 g single dose) showed that combination therapy was slightly more effective than monotherapy in the killing of *N. gonorrhoeae* and suppressing emergence of zoliflodacin resistance. Accordingly, WHO F was eradicated by only 0.5 g single dose of zoliflodacin in combination with doxycycline, and WHO X and SE600/18 were both eradicated by a 2 g single dose of zoliflodacin in combination with doxycycline; no zoliflodacin-resistant populations occurred during the 7-day experiment when using this zoliflodacin dose. When using suboptimal (0.5–1 g) zoliflodacin doses together with doxycycline, gonococcal mutants with increased zoliflodacin MICs, due to GyrB D429N and the novel GyrB T472P, emerged, but both the mutants had an impaired biofitness. The present study shows the high efficacy of zoliflodacin plus doxycycline combination therapy using a dynamic HFIM that more accurately and comprehensively simulate gonococcal infection and their treatment, i.e., compared to static *in vitro* models, such as short-time checkerboard experiments or time-kill curve analysis. Based on our dynamic *in vitro* HFIM work, zoliflodacin plus doxycycline for the treatment of both gonorrhea and chlamydia can be an effective combination.

## Introduction

Treatment and control of gonorrhea is threatened by increasing antimicrobial resistance (AMR) in *Neisseria gonorrhoeae*. Resistance has evolved to all previously used classes of antibiotics recommended for treatment. During the recent decade, *in vitro* and clinical resistance to the last-line options for empirical treatment, ceftriaxone and particularly azithromycin, has begun to spread (Unemo and Shafer, 2014; Unemo et al., 2019; Unemo et al., 2020; Workowski et al., 2021; Day et al., 2022; Sánchez-Busó et al., 2022). Furthermore, the first treatment failure with dual-therapy, ceftriaxone and azithromycin, was reported in 2016 (Fifer et al., 2016), and the international spread of the ceftriaxone-resistant FC428 clone has been verified since 2015 (Nakayama et al., 2016; Golparian et al., 2018; Lahra et al., 2018; Poncin et al., 2019; Zhou et al., 2020; Day et al., 2022; Lin et al., 2022). Furthermore, the first strains with resistance to ceftriaxone combined with high-level azithromycin resistance were identified in 2018 (Whiley et al., 2018; Jennison et al., 2019) and 2022 (Golparian et al., 2022; Pleininger et al., 2022). To mitigate the risk that gonorrhea becomes untreatable, which might become a reality for many bacterial infections (Algammal et al., 2023), global surveillance of the spread and evolution of AMR needs to be improved. Additionally, an enhanced understanding of the pharmacokinetics (PK) and pharmacodynamics (PD) for the optimization of current treatments and the use of molecular assays for resistance/susceptibility-guided therapy are necessary (WHO, 2012; Seña et al., 2020). However, new treatment options will become essential, which has been powerfully stressed in the WHO Global Action Plan: To Control the Spread and Impact of Antimicrobial Resistance in *N. gonorrhoeae* (WHO, 2012) and in the corresponding response plans from ECDC and CDC (CDC, 2012; ECDC, 2019). However, only two novel antimicrobials, zoliflodacin (Taylor et al., 2018a) and gepotidacin (Taylor et al., 2018b), have reached a later clinical stage of development for uncomplicated gonorrhea treatment.

Zoliflodacin is a novel spiropyrimidinetrione inhibiting bacterial DNA synthesis through the inhibition of topoisomerase type IIA with a unique mode of binding to GyrB (Basarab et al., 2015; Kern et al., 2015). Evaluation of zoliflodacin as a novel treatment for uncomplicated gonorrhea was recently finalized in a global phase III randomized controlled clinical trial (RCT) (ClinicalTrials.gov Identifier: NCT03959527). In this phase III RCT, zoliflodacin 3 g single oral dose met the pre-specified statistical test for non-inferiority when compared to the internationally recommended treatment including ceftriaxone 500 mg single intramuscular dose plus azithromycin 1 g single oral dose (5.31%, 95% confidence interval 1.38%, 8.65%). Non-inferiority of zoliflodacin was demonstrated within the pre-specified 12% margin and, additionally, within the 10% margin specified by the United States Food and Drug Administration (https://gardp.org/positive-results-announced-in-largest-pivotal-phase-3-trial-of-a-first-in-class-oral-antibiotic-to-treat-uncomplicated-gonorrhoea/). Zoliflodacin previously showed promising results in a phase II RCT (Taylor et al., 2018a), and additionally, the PD of zoliflodacin has been evaluated in a dynamic hollow-fiber infection model (HFIM) to identify the optimal zoliflodacin dosing, i.e., for ideal killing of *N. gonorrhoeae* and to suppress the selection of zoliflodacin resistance during treatment (Jacobsson et al., 2021; Jacobsson et al., 2022a). These HFIM studies showed that a ≥3 g single oral dose of zoliflodacin is required to successfully eradicate *N. gonorrhoeae* strains harboring pre-existing GyrB mutations that are predisposed to the development of zoliflodacin resistance (Jacobsson et al., 2021; Jacobsson et al., 2022a).

Doxycycline is a second-generation tetracycline, which is bacteriostatic through disruption of bacterial protein synthesis by binding to the 30S ribosomal subunit (Suzuka et al., 1966). Doxycycline is the first-line treatment for chlamydia, a first-line option for empiric treatment of non-gonococcal urethritis and cervicitis, used for initial empiric treatment in resistance-guided sequential treatment for *Mycoplasma genitalium*, and an alternative regimen for the treatment of syphilis, especially for patients with penicillin hypersensitivity (Lanjouw et al., 2016; Janier et al., 2021; Workowski et al., 2021; Jensen et al., 2022). Furthermore, two recent studies evaluating doxycycline post-exposure prophylaxis (PEP), i.e., after a potential exposure to a sexually transmitted infection (STI), showed promising results by decreasing the incidences of STIs (especially syphilis and chlamydia) (Molina et al., 2018; Luetkemeyer et al., 2022). Subsequently, the San Francisco Public Health Unit has started to offer doxycycline PEP to men who have sex with men (MSM) and trans women at a higher risk for STIs (San Francisco Department of Public Health, 2022). Consequently, if zoliflodacin is approved for the treatment of gonorrhea, it is likely that zoliflodacin will frequently be given together with doxycycline, i.e., because these other bacterial STIs are frequent coinfections to gonorrhea (or may not have been excluded), and the use of doxycycline PEP (Molina et al., 2018; Luetkemeyer et al., 2022) might quickly expand. It is crucial that doxycycline will then not inhibit the *N. gonorrhoeae* killing of zoliflodacin. It is a concern that, in a previous *in vitro* study, doxycycline/tetracycline significantly decreased zoliflodacin bactericidal activity (kill rate) in a 6-h time-kill curve analysis (Foerster et al., 2019). However, it was also stated as important to appropriately and quality assuredly evaluate the potential interactions between zoliflodacin and doxycycline/tetracycline in a dynamic HFIM (Foerster et al., 2019).

The objectives of this study were to evaluate different combination therapies with zoliflodacin (0.5–4 g in single oral doses) plus doxycycline (100 mg twice a day, for 7 days) against *N. gonorrhoeae* in our dynamic HFIM and compare with the previous results using zoliflodacin monotherapy (0.5–8 g) in HFIM (Jacobsson et al., 2021; Jacobsson et al., 2022a) for killing of *N. gonorrhoeae* and suppression of emergence of zoliflodacin resistance. The biofitness of the selected mutant WHO X-T472P, including the novel GyrB T472P substitution that increased zoliflodacin MIC, compared to its zoliflodacin-susceptible parental strain (WHO X) was also evaluated in HFIM.

## Material and methods

### Bacterial strains

The *N. gonorrhoeae* international reference strains WHO F (susceptible to relevant therapeutical antimicrobials) and WHO X (extensively drug-resistant, including resistance to ceftriaxone and other extended-spectrum cephalosporins and fluoroquinolones; zoliflodacin-susceptible) (Unemo et al., 2016), and the clinical *N. gonorrhoeae* strain SE600/18 (zoliflodacin-susceptible with GyrB S467N substitution, cultured in Sweden in 2018) (Jacobsson et al., 2022a) (Table 1) were examined.

**TABLE 1:** Phenotypic and genomic characteristics of investigated *N. gonorrhoeae* strains.

Antimicrobial tested	WHO F (Unemo et al., 2016)	WHO X (Unemo et al., 2016)	SE600/18 (Jacobsson et al., 2022a)
Strain genotype			
Zoliflodacin agar dilution MIC (microbroth MIC)^a^	0.064 (0.125)	0.125 (0.25)	0.25 (0.5)
Ceftriaxone (MIC)^a^	<0.002	2	0.032
Cefixime (MIC)^a^	<0.016	4	0.125
Ciprofloxacin (MIC)^a^	0.004	>32	0.5
Azithromycin (MIC)^a^	0.125	0.5	0.125
Doxycycline (MIC)^a^	0.25	4	2
Tetracycline (MIC)^a^	0.25	2	1
GyrB D429, K450, and S467	WT	WT	S467N
Gyr AS91 and D95	WT	S91F and D95N	S91F and D95N
*mtrR* promoter region 13 bp inverted repeat	WT	Deletion of A	WT
*mtrR*-coding region	WT	WT	WT
Mosaic *mtrRCDE*	-	-	-
PorB1b G120 and A121	NA	G120K and A121D	A121D
NG-MAST	ST3303	ST4220	ST20643
NG-STAR	ST2	ST226	ST3537
MLST	ST10934	ST7363	ST7363

MIC, minimum inhibitory concentration; WT, wild type; NA, not applicable; NG-MAST, *N. gonorrhoeae* multiantigen sequence typing; ST, sequence type; NG-STAR, *N. gonorrhoeae* sequence typing antimicrobial resistance; MLST, multi-locus sequence typing.

^a^ MIC (mg/L) of zoliflodacin was identified with agar dilution and microbroth methods, and MICs (mg/L) of ceftriaxone, cefixime, ciprofloxacin, azithromycin, doxycycline, and tetracycline, using ETEST (bioMérieux, Marcy-l’Etoile, France).

### Antimicrobial susceptibility testing

Zoliflodacin MICs (mg/L) were determined by agar dilution, according to Clinical and Laboratory Standards Institute (CLSI) guidelines (M07-A9 and M100-S24; www.clsi.org), using GCVIT agar plates [3.6% Difco GC Medium Base agar (BD, Diagnostics, Sparks, MD, United States) supplemented with 1% IsoVitaleX (BD, Diagnostics)] and microbroth dilution [in triplicates in the medium used in the HFIM medium, i.e., modified Fastidious Broth (mFB)], as previously reported (Jacobsson et al., 2021; Jacobsson et al., 2022a). MICs (mg/L) of doxycycline, tetracycline, ceftriaxone, cefixime, azithromycin, and ciprofloxacin were determined with ETEST, in concordance with the instructions of the manufacturer (bioMérieux, Marcy-l’Etoile, France).

### Hollow-fiber infection model

*N. gonorrhoeae* infections and PK/PD of therapeutic antimicrobials against *N. gonorrhoeae* were simulated using our dynamic HFIM with cellulosic cartridges (FiberCell Systems Inc., Frederick, MD, United States) (Jacobsson et al., 2021; Jacobsson et al., 2022a; Jacobsson et al., 2022b). A schematic drawing summarizing our HFIM can be seen in Supplementary Figure S1, and for more details regarding HFIM and two compartment models, see Cadwell (2012).

Briefly, zoliflodacin was co-administered with doxycycline to HFIM using syringe pumps, and peristaltic pumps isovolumetrically replaced the antibiotic-containing broth medium with the antibiotic-free medium to simulate different half-lives (t_1/2_) in the plasma of the two antibiotics and free (protein-unbound fraction) concentration-time profiles. Sampling for quantitative cultures (colony-forming units (CFUs)/mL) for total gonococcal burden and possible zoliflodacin-resistant and/or doxycycline-resistant gonococcal population, and the measurement of zoliflodacin and doxycycline concentrations were performed over 7 days. On the first day, 0.5 mL of gonococcal cultures (18–24 h) from GCAGP agar plates (3.6% Difco GC Medium Base agar (BD, Diagnostics) supplemented with 1% hemoglobin (BD, Diagnostics), 1% IsoVitaleX (BD, Diagnostics), and 10% horse serum) were inoculated in 49.5 mL of mFB and incubated at 36°C in a humidified 5% CO_2_-enriched atmosphere to a mid-log phase. A measure of 10 mL (∼10^5^–10^6^ CFUs/mL) of the gonococcal suspension was then inoculated into each HFIM cartridge to mimic a clinically relevant gonococcal load (Bissessor et al., 2011; Chow et al., 2016; Priest et al., 2017; Van Der Veer et al., 2020). Zoliflodacin was administered to simulate a PK plasma concentration-time profile in a human adult, following a zoliflodacin single oral dose (PK parameters for zoliflodacin 3 g oral dose were used: 17% fraction of free zoliflodacin in plasma, 6.47 h t_1/2_, and a 3-h infusion time, and were linearly adjusted for other doses) (O’Donnell et al., 2019), as previously described (Jacobsson et al., 2021; Jacobsson et al., 2022a). Doxycycline was administered to mimic an adult human PK plasma concentration-time profile, following a 100-mg oral dose of doxycycline twice a day for 7 days (PK parameters for doxycycline 100 mg oral dose were used: 15% fraction of free doxycycline in plasma, 17.1 h t_1/2_, and a 2-h infusion time) (Maesen et al., 1989; Nguyen et al., 1989; Alsarra et al., 2004; Gschwend et al., 2007; Binh et al., 2009). One HFIM cartridge for each investigated strain and experiment was used as a control of untreated growth.

Dose–range experiments (*n* = 2) were conducted simulating combination therapy with a single oral dose of 0.5, 1, 2, 3, or 4 g of zoliflodacin combined with a daily dose of 200 mg of doxycycline administered as 100 mg given at 0 h and at 12 h (q12 h) for 7 days, i.e., the recommended first-line treatment for *Chlamydia trachomatis* infections (Lanjouw et al., 2016; Workowski et al., 2021).

### Quantification of viable bacterial populations

To determine the *N. gonorrhoeae* total population and potentially zoliflodacin-resistant subpopulations, bacterial samples (1 mL) were taken from the extra capillary space of each cartridge at time points 2, 3, 6.5, 12, 14, 24, 48, 72, 96, 120, 144, and 168 h. Samples were serially diluted in mFB and plated in a quantitative manner on GCAGP agar plates and GCAGP agar plates containing 2–3×MIC of zoliflodacin, which resulted in a detection limit of ≥100 CFUs per HFIM cartridge, as previously reported (Jacobsson et al., 2021; Jacobsson et al., 2022a). Colony counts (log_10_ CFUs/mL) were conducted subsequent to incubation for up to 72 h at 36°C in a humidified 5% CO_2_-enriched atmosphere using an automated colony counter (Scan 4000, INTERSCIENCE, Saint-Nom-la-Bretèche, France).

### Biofitness experiments

To evaluate the biofitness of the mutant with increased zoliflodacin MICs selected in HFIM (WHO X-T472P) in comparison to the zoliflodacin-susceptible parental strain (WHO X), competition experiments using the coculture of WHO X-T472P and WHO X were performed in the HFIM, as previously described for the zoliflodacin-resistant mutant selected and its parental strain, i.e., SE600/18-D429N and SE600/18 (Jacobsson et al., 2022a; Jacobsson et al., 2022b). Briefly, gonococcal culture was collected from GCAGP agar plates and diluted in mFB to a quantity of ∼10^5^–10^6^ CFU/mL. Equal volumes (5 mL/strain) of the suspensions of each strain were inoculated into the identical HFIM cartridge. Aliquots (1 mL) were sampled at 24, 48, 72, 96, 120, 144, and 168 h, diluted in mFB in a serial manner and quantitatively plated on GCAGP agar plates and GCAGP agar plates containing 2×MIC of zoliflodacin, as previously reported (Jacobsson et al., 2022a; Jacobsson et al., 2022b). Colony counts (log10 CFU/mL) were conducted subsequent to incubation for up to 72 h at 36°C in a humidified 5% CO_2_-enriched atmosphere using an automated colony counter (Scan 4000, INTERSCIENCE, Saint-Nom-la-Bretèche, France). The competitive index (CI) was calculated by dividing the ratio of the mutant with increased zoliflodacin MIC, WHO X-T472P, to its zoliflodacin-susceptible parental strain, WHO X, at each time point with the ratio of the mutant to its wild-type parent strain in the initial inoculum (Vincent et al., 2019).

### Zoliflodacin and doxycycline concentration determination

To verify that the predicted zoliflodacin and doxycycline PK profiles were observed in HFIM, broth specimens (500 µL) were sampled at time points 1, 2, 3, 6.5, 12, 14, 24, 26, 48, 50, 72, 74, 96, 98, 120, 122, 144, 146, and 168 h. Concentrations of zoliflodacin were determined from 100-μL sample aliquots by liquid chromatography–tandem mass spectrometry (LC–MS/MS), as previously reported (Jacobsson et al., 2021; Jacobsson et al., 2022a; Jacobsson et al., 2022b). Concentrations of doxycycline were determined from 100-μL sample aliquots by LCMS/MS with isotopically labeled doxycycline as the internal standard.

### Comparative genomic analysis

Whole-genome sequencing (WGS) was conducted on colonies that were growing on the zoliflodacin-containing plates and that additionally exhibited enhanced zoliflodacin MICs as determined by agar dilution. The aim was to identify *gyrB* mutations associated with the increased MICs of zoliflodacin (Alm et al., 2015; Foerster et al., 2015; Foerster et al., 2019; Jacobsson et al., 2021; Jacobsson et al., 2022a). The WGS procedure followed the previously reported methodology (Jacobsson et al., 2016; Golparian et al., 2020). Briefly, all reads underwent quality control and trimming procedures, following the established CLC Genomics Workbench v20.0.4 workflow (Golparian et al., 2022). Subsequently, the quality-controlled reads were aligned to the WHO X reference genome obtained from Genbank (Accession: NZ_LT592155.1) using local alignment in CLC Genomics Workbench. The alignment employed a match score of 1, a mismatch cost of 2, and a linear gap cost of 3. Variants across the gene were identified with a minimum coverage of ×10 and a minimum frequency of 35%.

Additionally, the genomic sequences of the mutants with increased zoliflodacin MICs were investigated using the same procedure to identify or exclude other resistance-associated mutations selected in HFIM.

Raw reads associated with this study are accessible through the European Nucleotide Archive (ENA) with the accession number PRJEB64051.

## Results

### Phenotypic and genomic characteristics of investigated *N. gonorrhoeae* strains

In all examined strains, the MICs of zoliflodacin were similar (only one MIC doubling dilution higher) using the microbroth dilution method in comparison to the agar dilution method. The MICs of zoliflodacin and additional tested antimicrobials, and relevant genomic characteristics of the investigated gonococcal strains WHO F (susceptible to relevant therapeutical antimicrobials), WHO X (extensively drug-resistant; zoliflodacin-susceptible), and SE600/18 (zoliflodacin-susceptible with GyrB S467N substitution) are summarized in Table 1.

### Pharmacodynamic interaction of zoliflodacin and doxycycline in the hollow-fiber infection model

**FIGURE 1:**
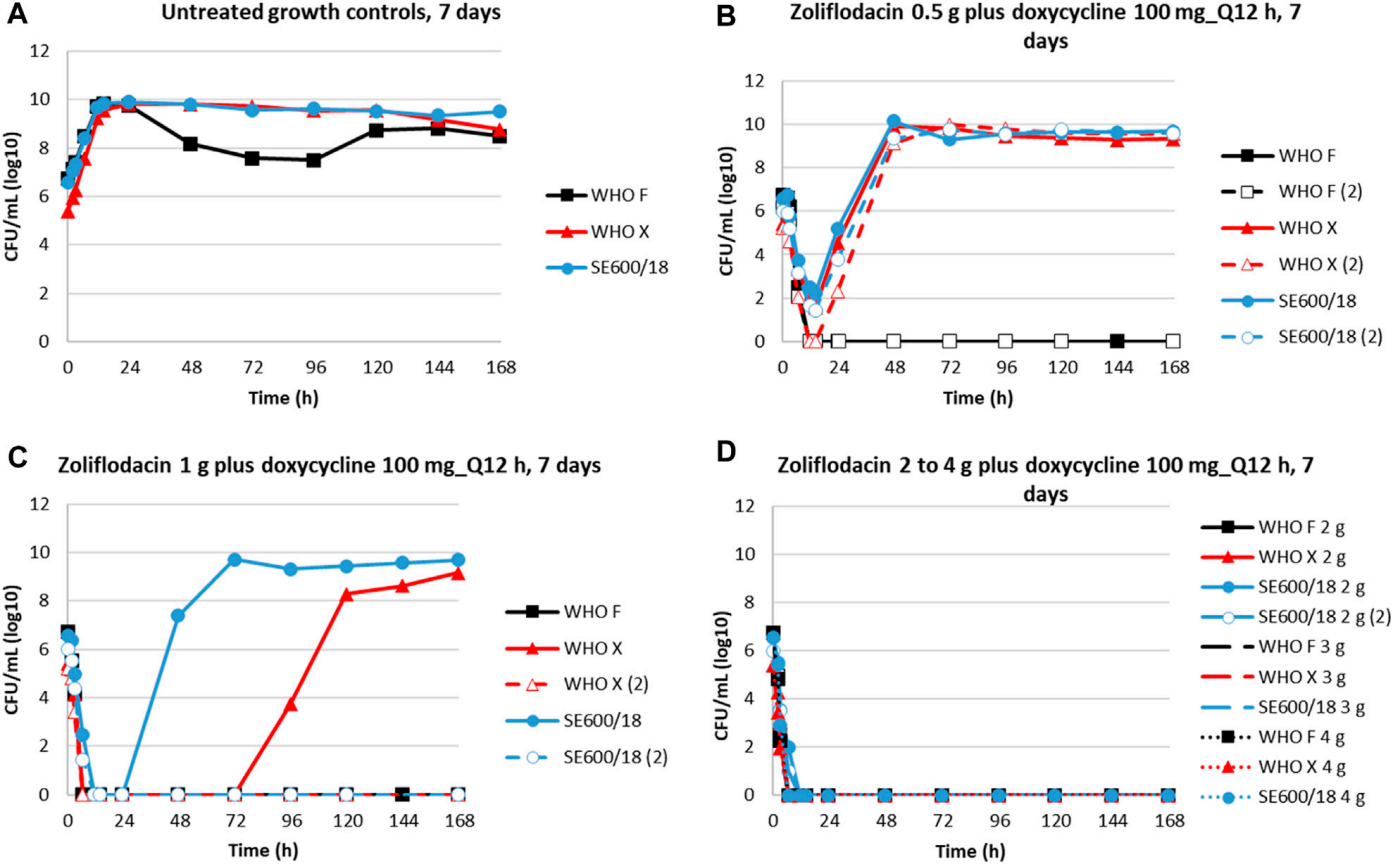
Growth curves of the total population of three *N. gonorrhoeae* strains (WHO F, WHO X, and SE600/18) in dose–range hollow-fiber infection Model (HFIM) experiments (performed in duplicate) showing untreated controls **(A)** and simulations of zoliflodacin single oral dose of 0.5 g **(B)**, 1 g **(C)**, 2 g, 3 g, and 4 g **(D)** combined with a daily dose of 200 mg doxycycline administered as 100 mg twice a day (Q12 h) for 7 days. HFIM experiments were monitored for 7 days.

HFIM dose–range experiments were performed to simulate the combination therapy of a single oral dose of 0.5–4 g of zoliflodacin combined with a daily oral dose of 200 mg doxycycline administered as 100 mg twice a day for 7 days (Figure 1). The untreated growth controls all grew well for the three examined strains obtaining a bacterial density of 10^9^–10^10^ CFUs/mL, except for a temporary decrease in growth at days 2–4 for WHO F, throughout the experiments lasting for 7 days (Figure 1A). The coadministration of zoliflodacin and doxycycline resulted in a rapid initial bacterial kill in all experiments. After a single 0.5 g zoliflodacin dose combined with the doxycycline regimen, WHO F was killed after 12 h in both conducted experiments, while WHO X and SE600/18 recovered and regrew to approximately 10^9^–10^10^ CFUs/mL at the 48-h time point (Figure 1B). When simulating a 1 g zoliflodacin dose combined with the doxycycline regime, WHO F was eradicated after 6.5 h, and WHO X and SE600/18 were both eradicated in one experiment and regrew in the other experiment reaching a bacterial density of 10^8^–10^10^ CFUs/mL after 120 h and 72 h, respectively (Figure 1C). Doses of 2–4 g zoliflodacin combined with the doxycycline regimen successfully eradicated all three strains within 12 h (Figure 1D).

On the zoliflodacin-containing agar plates, colonies were observed for WHO X and SE600/18 in the experimental arms of 0.5 g zoliflodacin combined with doxycycline and for SE600/18 also in the 1 g zoliflodacin combined with doxycycline arm. The WHO X colonies with a zoliflodacin MIC = 0.5 mg/L by agar dilution (parental strain MIC = 0.125 mg/L) harbored a GyrB T472P alteration, which has not been previously recognized to influence the MIC of zoliflodacin. The SE600/18 colonies displayed zoliflodacin MICs of 1–2 mg/L (parental strain MIC = 0.25 mg/L) and harbored the pre-existing GyrB S467N mutation plus a GyrB D429N alteration selected in the HFIM experiment, which has been previously shown to increase not only the zoliflodacin MIC but also to impair the growth and biofitness of the strain (Alm et al., 2015; Foerster et al., 2015; Foerster et al., 2019; Jacobsson et al., 2021; Jacobsson et al., 2022a).

**FIGURE 2:**
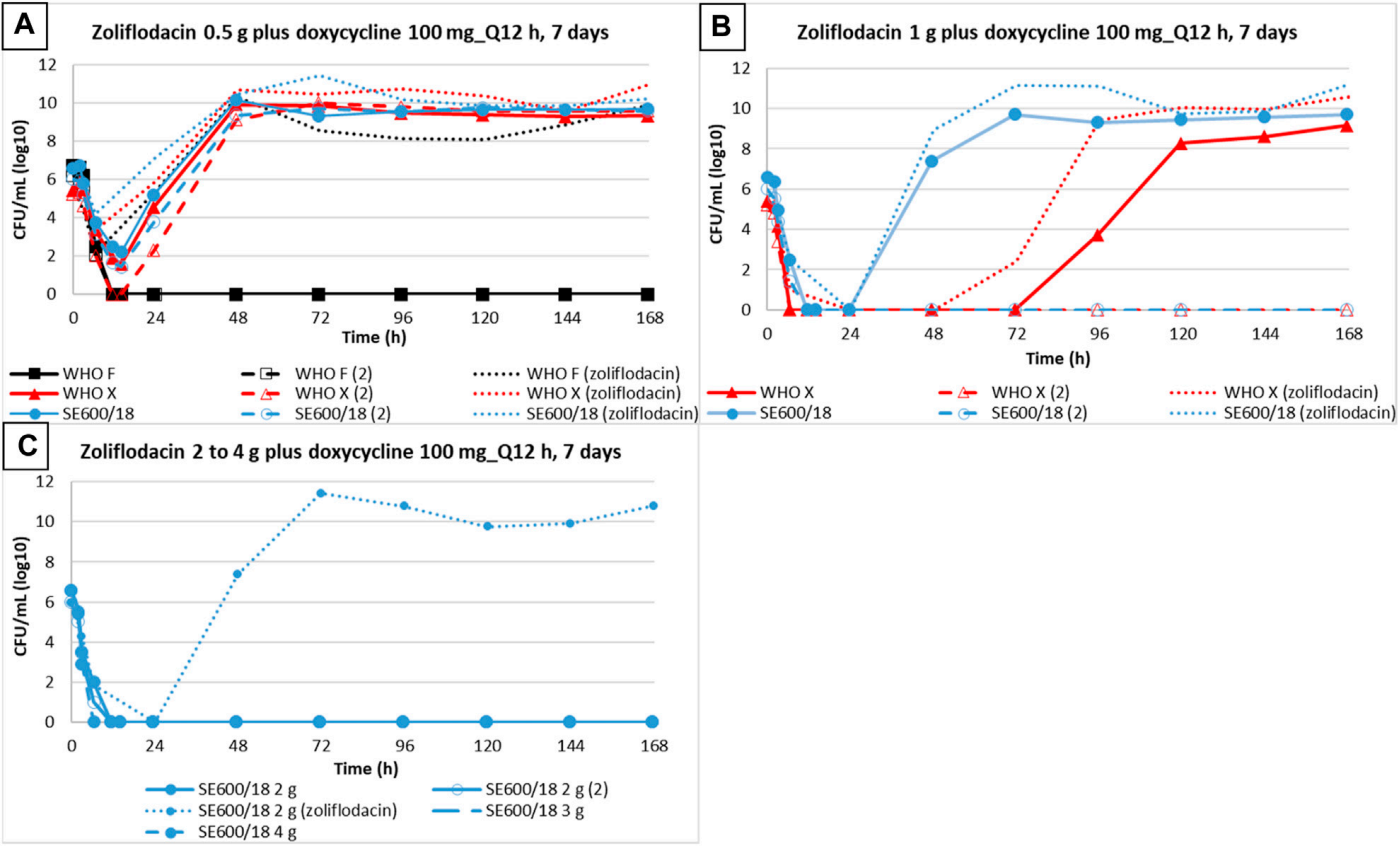
Growth curves of the total population of three *N. gonorrhoeae* strains (WHO F, WHO X, and SE600/18) simulating dual therapy with doxycycline (100 mg twice a day for 7 days) plus zoliflodacin 0.5 g **(A)**, 1 g, **(B)** 2 g, 3 g, and 4 g **(C)** are represented with solid and dashed lines. Dotted lines depict results from zoliflodacin monotherapy (Jacobsson et al., 2021; Jacobsson et al., 2022a).

When comparing *N. gonorrhoeae* eradication using dual therapy with the zoliflodacin–doxycycline regimen to our previous zoliflodacin monotherapy HFIM studies (Jacobsson et al., 2021; Jacobsson et al., 2022a) (Figure 2), we observed that 0.5 g zoliflodacin monotherapy failed to eradicate all three strains (Jacobsson et al., 2021; Jacobsson et al., 2022a), while WHO F was eradicated using the coadministration of zoliflodacin 0.5 g plus doxycycline in the present study. With 1 g zoliflodacin monotherapy, both WHO X and SE600/18 regrew and reached a bacterial density of 10^8^ CFUs/mL 24 h previously compared to one of the two HFIM experiments with zoliflodacin 1 g combined with doxycycline. In the second HFIM experiment with zoliflodacin and 1 g doxycycline, both WHO X and SE600/18 were eradicated. Finally, SE600/18 also regrew in 2 g zoliflodacin monotherapy (Jacobsson et al., 2021; Jacobsson et al., 2022a), whereas it was eradicated by zoliflodacin 2 g plus doxycycline dual therapy. Accordingly, zoliflodacin–doxycycline dual therapy was slightly more effective in gonococcal killing compared to zoliflodacin monotherapy (Figure 2).

**FIGURE 3:**
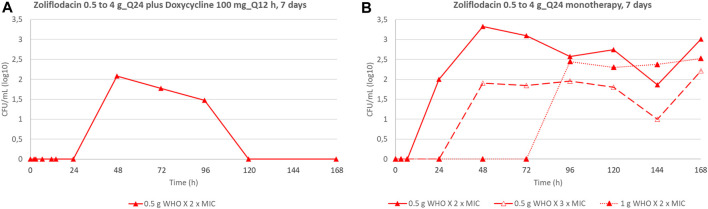
Total growth of populations with increased zoliflodacin MIC of the *N. gonorrhoeae* strain WHO X on the zoliflodacin-containing agar plates (2×MIC or 3×MIC) in the current experiments simulating dual-therapy with oral zoliflodacin and doxycycline (100 mg twice a day for 7 days) **(A)**, compared to our previous studies evaluating zoliflodacin monotherapy (Jacobsson et al., 2021) **(B)**.

We also compared the emergence of *N. gonorrhoeae* populations with increased zoliflodacin MICs by dual therapy with the zoliflodacin–doxycycline regimen to our previous zoliflodacin monotherapy HFIM studies (Jacobsson et al., 2021; Jacobsson et al., 2022a) (Figure 3). In brief, exposure of WHO X to both 0.5 g and 1 g zoliflodacin monotherapy for WHO X (Jacobsson et al., 2021) selected zoliflodacin-resistant GyrB D429N-containing *N. gonorrhoeae* mutants that were maintained until the end of the 7-day experiment (Figure 3B). However, only the zoliflodacin 0.5 g dose selected mutants with increased zoliflodacin MICs (with GyrB T472P causing the zoliflodacin MIC to increase from 0.125 mg/L to 0.5 mg/L) when combined with the doxycycline regimen, and the mutants were not detected after 96 h (Figure 3A) This indicates that the mutants were outcompeted by WHO X wild-type cells due to a decreased biofitness. Similar suppression of the emergence of zoliflodacin-resistant mutants with adequate biofitness was observed for SE600/18 (data not shown).

### Competition biofitness experiments (coculture) in HFIM

**FIGURE 4:**
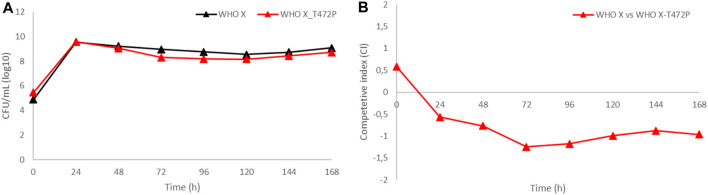
**(A)** Growth curves of the total population of the zoliflodacin-susceptible *N. gonorrhoeae* WHO X reference strain (black line) and the *in vitro*-selected WHO X-T472P mutant with increased zoliflodacin MIC (red line) when cocultured in the identical hollow-fiber infection model (HFIM) cartridge and monitored for 7 days. **(B)** Competitive index for the zoliflodacin-susceptible *N. gonorrhoeae* WHO X reference strain and the *in vitro*-selected WHO X-T472P mutant with increased zoliflodacin MIC, when cocultured in the identical HFIM cartridge and monitored for 7 days.

WHO X and the *in vitro*-selected WHO X-T472P mutant with increased zoliflodacin MICs were cocultured in the same HFIM cartridge for 7 days to investigate if the WHO X-T472P mutant selected *in vitro* displayed any altered growth and biofitness (Figure 4). The growth of both zoliflodacin-susceptible WHO X and WHO X-T472P with increased zoliflodacin MICs was maintained at approximately 10^8^–10^10^ CFUs/mL during the experiment lasting for 7 days, i.e., similar to the bacterial density when monocultured (Figure 4A). However, the calculated competitive index (Figure 4B) displayed an impaired biofitness, especially during the first 72 h, of WHO X-T472P with increased zoliflodacin MIC compared to the zoliflodacin-susceptible WHO X parent strain. The HFIM experiments of the WHO F, WHO X, and SE600/18 strains and their results have been summarized in Supplementary Figure S2.

## Discussion

Gonorrhea remains an important public health concern with 82 million new cases estimated among persons aged 15–49 years in 2020 globally (WHO, 2021). The high incidence and prevalence of infection combined with factors like its extraordinary capacity to develop resistance to all classes of antimicrobials introduced for treatment, the large number of asymptomatic infections, which constitutes a reservoir of infection, and the cultural stigma associated with the infection in some parts of the world contribute to the urgent need for improvement of the management and control of gonorrhea. Zoliflodacin, the first-in-class spiropyrimidinetrione, is bactericidal against *N. gonorrhoeae* with a low *in vitro* frequency of resistance and high antibacterial activity with an MIC_90_ value of 0.125 mg/L when global, including multi- and extensively drug-resistant, clinical *N. gonorrhoeae* isolates from the recent decades have been investigated (Jacobsson et al., 2014; Unemo et al., 2015; Bradford et al., 2020; Le et al., 2021; Unemo et al., 2021; Berçot et al., 2022; Pleininger et al., 2022). Results from the conducted phase II RCT using 3 g single oral dose of zoliflodacin demonstrated 100% microbiological cure for urogenital and rectal gonorrhea and 78% for pharyngeal gonorrhea, and no zoliflodacin-resistant gonococcal isolates were found (Taylor et al., 2018a). Furthermore, by using our dynamic HFIM for gonorrhea, we have previously demonstrated that to provide both effective killing of *N. gonorrhoeae* and suppression of the selection of zoliflodacin resistance, zoliflodacin should be given as a ≥3 g single dose. This dose also targets the rare zoliflodacin-susceptible *N. gonorrhoeae* strains harboring a pre-existing zoliflodacin-target GyrB S467N substitution which predisposes for the development of zoliflodacin resistance by a second-step mutation, i.e., GyrB D429N (Jacobsson et al., 2021; Jacobsson et al., 2022a; Golparian et al., 2022).

If zoliflodacin is approved for the treatment of gonorrhea, it is likely to be administered with doxycycline because other bacterial STIs, such as chlamydia, syphilis, or *M. genitalium* infections, are frequently diagnosed coinfections with gonorrhea (or may not have been excluded). Moreover, the use of doxycycline PEP (Molina et al., 2018; Luetkemeyer et al., 2022) will likely quickly expand. Consequently, the pharmacodynamic interaction between the two drugs was assessed in the present study. Zoliflodacin 0.5–4 g treatment in combination with the recommended 7-day doxycycline treatment regimen (100 mg twice a day for 7 days) was simulated in our dynamic *in vitro* HFIM. In brief, WHO F, susceptible to all relevant antimicrobials, was eradicated when simulating a single 0.5 g zoliflodacin dose combined with the doxycycline regimen. Both WHO X, extensively drug-resistant, including all extended-spectrum cephalosporins and fluoroquinolones, as well as SE600/18, a clinical zoliflodacin-susceptible *N. gonorrhoeae* strain with GyrB S467N substitution, were eradicated in one experiment simulating a single 1 g zoliflodacin dose combined with the doxycycline regimen, and recovered and regrew in one experiment. In the experiment where WHO X regrew, the novel GyrB T472P substitution, resulting in an increase in the zoliflodacin MIC from 0.125 mg/L to 0.5 mg/L, was identified. Notably, the WHO X-T472P mutant displayed a slightly decreased biofitness, which was observed by its eradication after 92 h in the HFIM experiment and in competitive coculture experiments. In the resistant populations of SE600/18, we identified the previously described GyrB D429N substitution that has been shown to increase the MICs of zoliflodacin (Alm et al., 2015; Foerster et al., 2015; Foerster et al., 2019; Jacobsson et al., 2021; Jacobsson et al., 2022a). However, compared to zoliflodacin monotherapy (Jacobsson et al., 2021; Jacobsson et al., 2022a), zoliflodacin plus doxycycline dual therapy further suppressed the emergence of zoliflodacin resistance, which only emerged when suboptimal doses of <3 g zoliflodacin were used. Consequently, adding doxycycline to the zoliflodacin treatment seemed advantageous for both the bacterial kill rate and resistance suppression compared to our previous studies, mimicking zoliflodacin monotherapy. With dual therapy, all three examined strains were eradicated by a lower zoliflodacin dose (WHO F 0.5 g compared to 1 g and SE600/18 2 g compared to a 3 g zoliflodacin dose) or the complete regrowth was observed later during the experiment (120 h compared to 96 h for WHO X using 1 g zoliflodacin dose). These results are in contrast to the results from a previous static *in vitro* study (Foerster et al., 2019), in which it was indicated that doxycycline/tetracycline inhibits the gonococcal killing of zoliflodacin in 6-h time-kill curve analysis. Accordingly, in the present study, we show the importance of using dynamic HFIM that more accurately and comprehensively simulate gonococcal infections and their treatment, i.e., compared to static *in vitro* models, such as short-time checkerboard experiments or time-kill curve analysis, which should be interpreted with caution.

Dual antimicrobial therapy can be a way to preserve an antibiotic and to prolong its usefulness; however, to evaluate if the combination of antibiotics is beneficial, if there is a synergistic effect or, at a minimum, an additive interaction so that the bacterial killing is enhanced and the likelihood of suppression of resistance is increased, various properties of the drug need to be considered. HFIM allows to alter the antimicrobial concentration, simulate various dosing regimens and different distribution schemes, single or multiple doses, and also simulate and combine antibiotics with differences in half-life, and all at the same time monitoring the efficacy in bacterial killing and the development of resistant subpopulations during treatment under the different administration schemes all to evaluate the best overall treatment effect (Nielsen and Friberg, 2013; Lodise et al., 2020; Hou et el., 2022). There are limitations linked to HFIM to be considered when interpreting these findings. For example, the efficacy of a specific drug represents a conservative estimate as the model does not account for the immune response of the host and therefore does not fully reflect the human response to bacteria and antibiotics. Furthermore, a limitation coupled to the present study is the nonexistence of human PK data from the major gonococcal infection sites, i.e., the urogenital tract, the anogenital tract, and the oropharynx. However, this is a limitation in most similar STI studies, i.e., for the evaluation of novel antibiotics and for antimicrobials currently recommended for treatment (Kong et al., 2019). Consequently, the HFIM simulations for gonorrhea treatment were in the present study based on concentrations of free zoliflodacin and doxycycline in human plasma that may not correspond to the systemic exposures at the urogenital and extragenital infection sites in an ideal manner. Despite this, antimicrobial plasma concentrations are often used as surrogates for infection site concentrations as these are unknown and have shown to serve well in linking drug exposure to effect (Drusano, 2004).

In conclusion, by examining the pharmacodynamics of zoliflodacin in combination therapy with doxycycline against *N. gonorrhoeae* in our dynamic *in vitro* gonococcal HFIM, we demonstrated that combination therapy including zoliflodacin (0.5 g–4 g single dose) and doxycycline (200 mg divided into 100 mg twice a day for 7 days) displays a high efficacy in the *N. gonorrhoeae* killing rate and suppression of resistance emergence, i.e., an increased efficacy compared to zoliflodacin monotherapy (Jacobsson et al., 2021; Jacobsson et al., 2022a). The future use of zoliflodacin for the treatment of gonorrhea will require additional evaluations, including the investigation of antagonism or synergism with other antibiotics potentially co-administered for the treatment of gonorrhea, additional concomitant STIs, or other infectious diseases.

## Data Availability

The datasets presented in this study can be found in online repositories. The names of the repository/repositories and accession number(s) can be found here: European Nucleotide Archive (ENA) with the accession number PRJEB64051.

## Supplementary material

Supplementary Figure S1 Hollow fiber infection model (two compartment model). *Neisseria gonorrhoeae* cells replicate and are retained in the hollow fiber cellulosic cartridge. The central reservoir is continuously re-circulating the growth medium, and eliminates waste of the growth medium. Antibiotic is added in a controlled manner, associated with the examined dosing regimen, and the elimination kinetics are controlled by the addition of diluent to the central reservoir. The volume in the central reservoir is maintained constant.

Supplementary Figure S2 Schematic flow chart summarizing the hollow fiber infection model (HFIM) experiments of the WHO F, WHO X, and SE600/18 strains and their results. MIC, minimum inhibitory concentration.
