# Supplementary material for: Pharmacodynamics of zoliflodacin plus doxycycline combination therapy against Neisseria gonorrhoeae in a gonococcal hollow-fiber infection model
Source: Front Pharmacol. 2023 Dec 7;14:1291885. doi: 10.3389/fphar.2023.1291885 (PMC10733441; doi:10.3389/fphar.2023.1291885)
Supplement: Supplementary file 1 [file DataSheet1.pdf]

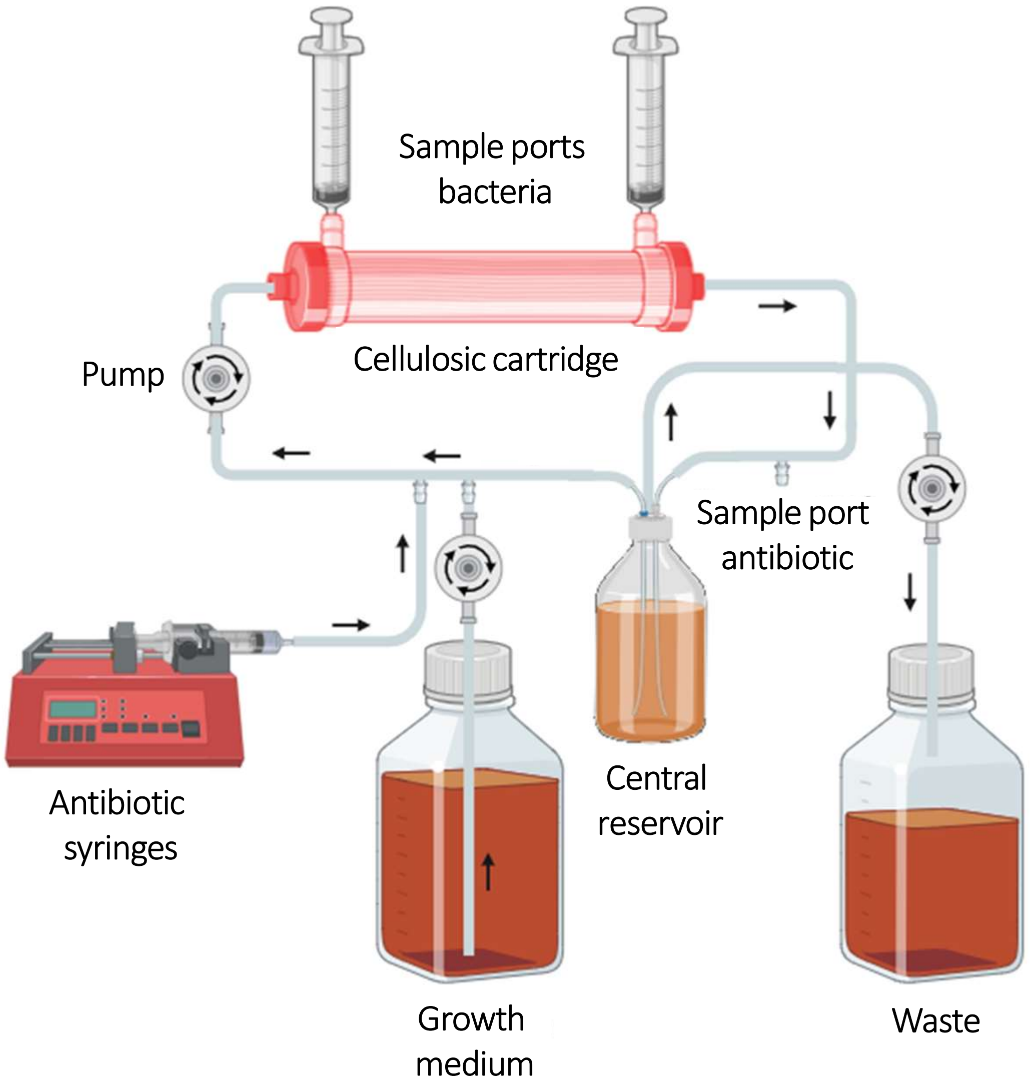
Sample ports
bacteria
Cellulosic cartridge
Pump
Sample port
antibiotic
Antibiotic
syringes
Central
reservoir
Growth
medium
Waste

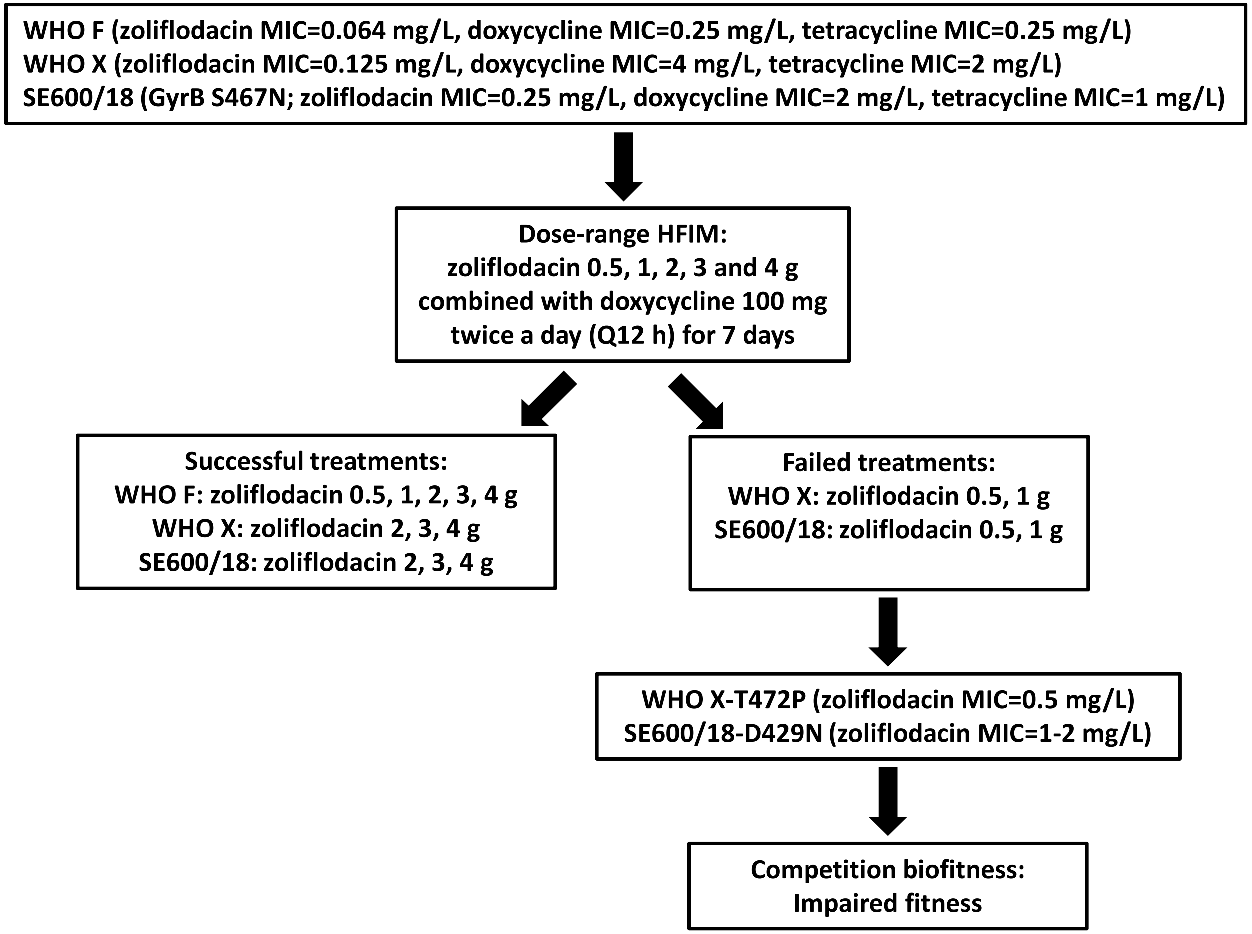
WHO F (zoliflodacin MIC=0.064 mg/L, doxycycline MIC=0.25 mg/L, tetracycline MIC=0.25 mg/L)
WHO X (zoliflodacin MIC=0.125 mg/L, doxycycline MIC=4 mg/L, tetracycline MIC=2 mg/L)
SE600/18 (GyrB S467N; zoliflodacin MIC=0.25 mg/L, doxycycline MIC=2 mg/L, tetracycline MIC=1 mg/L)
Dose-range HFIM:
zoliflodacin 0.5, 1, 2, 3 and 4 g
combined with doxycycline 100 mg
twice a day (Q12 h) for 7 days
Successful treatments:
WHO F: zoliflodacin 0.5, 1, 2, 3, 4 g
WHO X: zoliflodacin 2, 3, 4 g
SE600/18: zoliflodacin 2, 3, 4 g
Failed treatments:
WHO X: zoliflodacin 0.5, 1 g
SE600/18: zoliflodacin 0.5, 1 g
WHO X-T472P (zoliflodacin MIC=0.5 mg/L)
SE600/18-D429N (zoliflodacin MIC=1-2 mg/L)
Competition biofitness:
Impaired fitness
